# Study of transmission dynamics of novel COVID-19 by using mathematical model

**DOI:** 10.1186/s13662-020-02783-x

**Published:** 2020-07-01

**Authors:** Rahim Ud Din, Kamal Shah, Imtiaz Ahmad, Thabet Abdeljawad

**Affiliations:** 1 Department of Mathematical, University of Malakand, Dir(L), Khyber Pakhtunkhwa, Pakistan; 2 Department of Mathematics and General Sciences, Prince Sultan University, Riyadh, Saudi Arabia; 3 Department of Medical Research, China Medical University, Taichung, 40402 Taiwan; 4 Department of Computer Science and Information Engineering, Asia University, Taichung, Taiwan

**Keywords:** Mathematical model, Novel coronavirus-19, Nonstandard finite difference scheme, Immigration rate

## Abstract

In this research work, we present a mathematical model for novel coronavirus-19 infectious disease which consists of three different compartments: susceptible, infected, and recovered under convex incident rate involving immigration rate. We first derive the formulation of the model. Also, we give some qualitative aspects for the model including existence of equilibriums and its stability results by using various tools of nonlinear analysis. Then, by means of the nonstandard finite difference scheme (NSFD), we simulate the results for the data of Wuhan city against two different sets of values of immigration parameter. By means of simulation, we show how protection, exposure, death, and cure rates affect the susceptible, infected, and recovered population with the passage of time involving immigration. On the basis of simulation, we observe the dynamical behavior due to immigration of susceptible and infected classes or one of these two.

## Introduction

Recently, the whole world has been suffering due to a novel coronavirus pandemic. It was named novel coronavirus infectious disease (COVID-19) which was claimed to outbreak first in Wuhan, central China (see [1]). Novel coronavirus-19 is a new chain of corona group of viruses that had not been identified in human history before December 2019. For the first time COVID-19 was found in Wuhan, China in December 2019 and has spread to various urban areas in China as well as round about 196 different countries of the world. It has since been declared an outbreak by World Health Organization (WHO). According to the data reported by WHO (World Health Organization), on 11 June 2020, the reported laboratory confirmed that the number of affected humans reached more than 7.5 million including more than 0.425 million death cases recorded. Some researchers have also claimed that there are other sources of this corona virus including dogs, pangolin, etc. As per recoded data, the death rate is different in different countries. Currently the highest death rate has been observed in Europe, USA, and Iran. The number of confirmed cases has been growing on a very fast track on a daily basis, and it has been declared a worldwide pandemic disease.

On 31 December 2019, the WHO reported a novel corona virus (2019-nCoV) in Wuhan City, Hubei Province of China in humans, see [2, 3]. It was named severe acute respiratory syndrome coronavirus 2 (SARS-CoV-2) by the International Committee on Taxonomy of Viruses on 11 February 2020 (for details, we refer to [4–10]). Firstly, this outbreak was identified in Wuhan with most early cases being reported in the city and later spread to other countries at an alarming rate and became a lethal disease. There are different schools of thought behind the origin of COVID-19: some say that it might be of bat origin (see [11]), some say that it might be related to a seafood market exposure (see [12]). International travel of any form has been a potential reason for the fast spread of COVID-19 [2, 12–14]. So, immigration has a severe impact on the severity of spreading of COVID-19. It has been stated (fact) that the origin of novel COVID-19 is the transmission from animal to human as many infected cases claimed that they had been to a local fish and wild animal market in Wuhan in November [15]. Soon, some researchers confirmed that the transmission also happened person to person (see [2]). In the present situation this pandemic has produced a very harmful effect on health, economics, and social life of the whole globe. In the whole world researchers, policy makers, and doctors are struggling to control this serious pandemic so that the lives of maximum people may be secured. They observed this disease from their own point of view. Also it is a fact that most people infected with novel COVID-19 will experience mild to moderate respiratory illness. Common symptoms are fever, tiredness, dry cough, and throat infection. Some people may experience aches and pains, nasal congestion, runny nose, sore throat, diarrhoea, etc.

Since mathematical models are powerful tools to understand the dynamics of real world phenomena, particulary the transmission of an infectious disease, in literature large numbers of mathematical models of infectious disease have been studied, we refer to a few of them [16–22]. Also the area of modeling has been extended recently to noninteger order and nonlocal derivatives of fractional order [23, 24]. By using mathematical models for understanding the transmission dynamics of a disease can help the researchers to make future prediction and to adopt some precautionary measures to save maximum population from being lost. Also the mentioned tools help to make strategies to control or eliminate the disease from society. Same as the case of current novel COVID-19, which has been studied from different aspects in the last few months (for details, see [25–29]). Therefore, motivated by the aforesaid discussion, we observed that immigration has major roles in spreading the current disease in our society. It has been observed that due to immigration, this disease has spread in the whole globe within two to three months. Therefore in this work we construct a modified SIR type model involving immigration rate to investigate the transmission dynamics of the aforementioned disease. For numerical simulation, we use the nonstandard finite difference (NSFD) scheme. The concerned method is an efficient and powerful method to find numerical solutions to many nonlinear problems. Therefore various researchers have used this method for the numerical simulation of many bathetical models (for instance, see [17, 30] and the references therein).

## Model formulation

**Figure 1:**
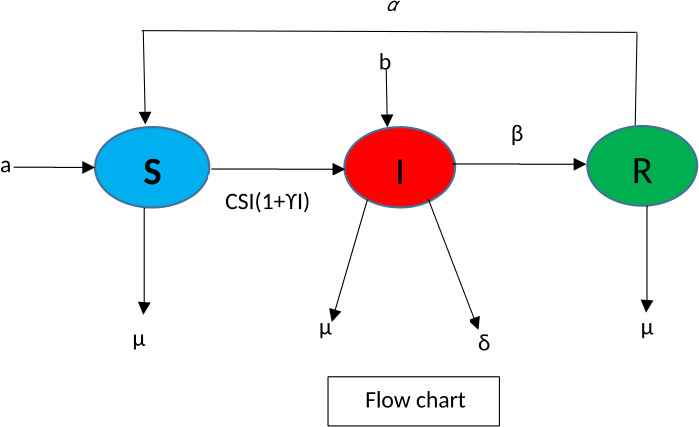
Flow chart of the model (1)

This part of the paper is devoted to constructing the mathematical model for our proposed problem. We take here three compartments: susceptible $S(t)$, infected $I(t)$, and recovered $R(t)$. We construct the required model under convex incidence rate which is assumed to be a convex function with respect to the infective class due to host population. The benefit of using convex incidence rate is that it corresponds to an increased rate of infection because of two exposures over a small time period: a single contact produces infection at the rate $CIS$, while the new infective individuals arise from double exposures with $CI^{2}S$. It produces further chance that the recovered individual again may catch infection. Here we remark that the function $\varPhi (S, I)=CI(t)S(t)(1+\gamma I(t))$, where both *C*, *γ* are positive constants. This is an interesting example for nonlinear incidence rate already used by some authors [17, 31, 32]. The flow chart of the model is shown in Fig. 1.

The dynamics of the population are described by the following differential equations:

1 $$ \begin{aligned} &\frac{dS(t)}{dt} =a -CI(t)S(t) \bigl(1+\gamma I(t)\bigr) -\mu S(t) + \alpha R(t), \\ &\frac{dI(t)}{dt} =CI(t)S(t) \bigl(1+\gamma I(t)\bigr)-(\beta +\mu +\delta -b)I(t), \\ &\frac{dR(t)}{dt} =\beta I(t) -(\alpha +\mu )R(t). \end{aligned} $$

The parameters involved in model (1) are described as in Table 1.

**Table 1:** The physical interpretation of the parameter

Parameters	The physical interpretation
*S*(*t*)	Susceptible compartment
*I*(*t*)	Infected compartment
*R*(*t*)	Recovered compartment
*a*	The recruitment rate
*μ*	Natural death
*δ*	Death due to corona
*b*	The immigration rate of infected individuals
*β*	Corona infection recovery rate
*C*	The infection rate
*γ*	Rate at which recovered individuals lose immunity
*α*	Rate of recovery from infection

**Table 2:** The physical interpretation of the parameters and numerical values

Parameters	Physical description	Numerical value	Reference
*S*(*t*)	Initial susceptible compartment	12.6 million	[26]
*I*(*t*)	Initial infected compartment	0.084 million	[26]
*R*(*t*)	Initial recovered compartment	0 million	[26]
*a*	The birth rate of infection	0.1243	[28]
*μ*	Natural death	0.002	[28]
*δ*	Death due to corona	0.05	[28]
*b*	The immigration rate rate	0.0205	[29]
*β*	Corona infection recovery rate	0.09871	[27]
*C*	Infection rate	0.580	[28]
*γ*	Rate at which recovered individuals lose immunity	0.0003	[29]
*α*	Rate of recovery	0.854302	[27]

First, for the equilibrium of model (1), we consider its existence. Corresponding to some values of parameters, there exists a disease-free equilibrium for system (1) denoted by $E_{0} = (a/\mu ,0, 0)$. To compute the nonnegative equilibrium, we have

$$\begin{aligned} \begin{aligned} & a -CI(t)S(t) \bigl(1+\gamma I(t)\bigr) -\mu S(t) + \alpha R(t)=0, \\ & CI(t)S(t) \bigl(1+\gamma I(t)\bigr)-(\beta +\mu +\delta -b)I(t)=0, \\ & \beta I(t) -(\alpha +\mu )R(t)=0. \end{aligned} \end{aligned}$$

To find the basic reproduction number $R_{0}$, let $x = (S(t), I(t))$ in model(1). Then

$$ \frac{dx}{dt}=\mathcal{F}-\mathcal{V}, $$

where

$$ \mathcal{F}= \begin{pmatrix} CI(t)S(t)(1+\gamma I(t)) \\ 0 \end{pmatrix} $$

and

$$ \mathcal{V}= \begin{pmatrix} (\mu -a)S(t) \\ (\beta +\mu +\delta -b)I(t) \end{pmatrix} $$

for the disease-free equilibrium Jacobian of $\mathcal{F}$ is

$$ F= \begin{pmatrix} 0 & CS^{0} \\ 0 & 0 \end{pmatrix} $$

and Jacobian of $\mathcal{V}$ to deduce the disease-free equilibrium is given by

$$ V= \begin{pmatrix} \mu -a & 0 \\ 0 & \beta +\mu +\delta -b \end{pmatrix}. $$

Hence, for model (1), by simple calculation, we have

$$ FV^{-}1= \begin{pmatrix} 0 & \frac{CS^{0}(\mu -a)}{\beta +\mu +\delta -b} \\ 0 & 0 \end{pmatrix}. $$

Hence the basic reproduction number (reproductive rate) $R_{0}$ is

2 $$ R_{0}=\frac{ac}{\mu (\beta +\mu +\delta -b)}. $$

From (2), we clearly observe that

(i) There are no positive equilibria of model (6) if $R_{0}\leq 1$;
(ii) A unique positive equilibrium also known as endemic equilibrium $E^{*}(t) = (S^{*}(t), I^{*}(t),R^{*}(t))$ exists under $R_{0}>1$.

The endemic equilibrium is given by

$$ \begin{aligned} & S^{*}(t)= \biggl( \frac{\alpha \beta }{CI^{*}(1+\gamma I^{*})+\mu -a} \biggr)I^{*}, \\ & I^{*}(t)= \frac{-(\mu -a)(\beta +\mu +\delta -b)+C \beta \gamma \alpha +\sqrt{\varOmega }}{2C \gamma (\beta +\mu +\delta -b)}, \\ & R^{*}(t)=\frac{\beta }{\alpha +\mu }I(t)^{*} . \end{aligned} $$

The value of *Ω* is given as

3 $$ \varOmega =(\mu -a) \bigl((\delta + \beta -b +d)+C \beta \gamma \alpha \bigr)^{2} -4\alpha \beta c^{2}(\mu -a) (\delta + \beta -b+\mu ). $$

Next, we will elaborate on the characteristics of these equilibria and a global mathematical analysis of system (1).

## Dynamical behavior of the model

To elaborate the dynamic of system (1), we have the following lemma.

### Lemma 1

*System* (1) *has invariant manifold of plane*$S(t)+I(t)+R(t)= a/\mu $, *which is fascinating in the* 1*st octant*.

### Proof

Add up all the equations of system (1) and let $N(t) = S(t) + I(t) + R(t)$. Then

4 $$ \begin{aligned} &\frac{dS(t)}{dt}+ \frac{dI(t)}{dt}+\frac{dR(t)}{dt}=a- \mu S(t)-\mu I(t)-\mu R(t)+bI(t)- \delta I(t), \\ &\frac{d}{dt}\bigl(S(t)+I(t)+R(t)\bigr)=a-(\delta -b)I(t)-\mu \bigl(S(t)+E(t)+I(t)+R(t)\bigr) \end{aligned} $$

(4) implies that

5 $$ \frac{dN(t)}{dt}=a-(\delta -b)I(t)-\mu N(t). $$

Hence for (5) we present the general solution as

$$ N(t)=\frac{1}{\mu } \bigl[a-(\delta -b)I(t)-dN(t_{0})e^{(t_{0}-t)} \bigr], $$

which completes our conclusion. □

Now we reduce system (1), because obviously the limit set of (1) on plane $S(t) + I(t) + R(t) = \frac{a}{\mu }$ has a limit set:

6 $$ \begin{aligned} &\frac{dI(t)}{dt}=C \biggl( \frac{a}{\mu }-I(t)-R(t) \biggr) \bigl(1+ \gamma I(t)\bigr)-(\beta +\mu + \delta -b)\stackrel{\Delta }{=} \omega\bigl(I(t),R(t)\bigr), \\ &\frac{dR(t)}{dt}=\beta I(t)-(\mu +\alpha )R(t)\stackrel{\Delta }{=} \xi \bigl(I(t),R(t)\bigr). \end{aligned} $$

We have the following theorem with regards to the nonexistence of cyclical shells in system (6), which shows the nonexistence of cyclical shells of system (1) by Lemma 1.

### Theorem 1

*There do not exist nontrivial periodic orbits corresponding to system* (6).

### Proof

Consider a “Dulac function” and consider system (6) for $I(t)>0$ and $R(t)>0$.

$$ D\bigl(I(t),R(t)\bigr)=\frac{1+\gamma I(t)}{CI(t)}. $$

Then we have

7 $$\begin{aligned}& D\omega = \biggl(\frac{a}{\mu }-I(t)-R(t) \biggr) \bigl(1+\gamma I(t) \bigr)- \frac{(\beta +\mu +\delta -b)(1+\gamma I(t))}{C}, \\& D\xi = \frac{\beta I(t)}{C} \bigl(1+\gamma I(t) \bigr)- \frac{(\mu +\alpha )(1+\gamma I(t))R(t)}{CI(t)}, \\& \begin{aligned} &\frac{\partial (D\omega )}{\partial I(t)}=-\bigl(1+\gamma I(t)\bigr) \biggl[1+2 \gamma R(t)+3 \gamma I(t)-\frac{2 \gamma a}{\mu } \biggr]- \frac{\gamma }{C} [\beta +\mu D-b ], \\ &\frac{\partial (D\xi )}{\partial R(t)}=- \frac{(\mu +\alpha )(1+\gamma I(t))}{CI(t)}. \end{aligned} \end{aligned}$$

By adding all equations of (7), we have

8 $$ \frac{\partial (D\omega )}{\partial I(t)}+ \frac{\partial (D\xi )}{\partial R(t)} =-\bigl(1+\gamma I(t)\bigr) \biggl[1+2 \gamma R(t)+3 \gamma I(t)-\frac{2 \gamma a}{\mu } \biggr]. $$

Hence

$$ -\frac{\gamma }{C} [\beta +\mu \delta -b ]- \frac{(\mu +\alpha )(1+\gamma I(t))}{CI(t)}< 0, $$

which proves the conclusion of the theorem. □

To study $S_{0}$ disease-free equilibrium and its properties, and also the endemic equilibrium $S^{*}$, we recall (6) with

$$\begin{aligned}& x = \frac{C}{\mu +\alpha }I(t), \\& y = \frac{C}{\mu +\alpha }R(t), \\& \tau = (\mu +\alpha )t. \end{aligned}$$

One can obtain from (6)

9 $$ \begin{aligned} &\frac{dx}{d\tau }= \frac{x}{1+qx}(B-x-y)-nx, \\ &\frac{dy}{d\tau }=px-y, \end{aligned} $$

with

$$\begin{aligned}& p = \frac{\beta }{\mu +\alpha }, \\& n = \frac{\beta +\mu +\delta -b}{\mu +\alpha }, \\& B = \frac{aC}{\mu (\mu +\alpha )}, \\& q = \frac{\gamma (\mu +\alpha )^{2}}{C^{2}}. \end{aligned}$$

### Note

Keep in mind that $(0, 0)$ may be obtained from system (9). In fact the disease-free equilibrium $S_{0}$ of system (1) and $(x^{*},y^{*})$ of system (9) is the unique positive equilibrium, which is, in fact, the endemic equilibrium $S^{*}$ of system (1) under the condition $n-B < 0$ with $x^{*}=\frac{B-n}{q-1}$ and $y^{*}=px^{*}$. At first glance, we investigate for $(0, 0)$ the stability and topological type trivial equilibrium. At the point $(0, 0)$, the Jacobian matrix of system (9) is given by

10 $$ M_{0}= \begin{pmatrix} B-n & 0 \\ p & -1 \end{pmatrix}. $$

The dynamic of system (9) is equivalent to (11). If $B-n=0$, then there exists a small neighborhood $N_{0}$ of $(0, 0)$.

11 $$ \begin{aligned} &\frac{dx}{d\tau } =-x-2y+O \bigl((x,y)^{3}\bigr), \\ &\frac{dy}{d\tau } =px-y. \end{aligned} $$

From (11) $(0, 0)$ is a saddle node. The next results is important.

### Theorem 2

*The trivial equilibrium point of system* (1) *possesses the following properties*:

(i) *As a result the system has a hyperbolic saddle if*$n< B$.
(ii) *As a result the system has a saddle node if*$n=B$.
(iii) *As a result the system has a stable hyperbolic node if*$n>B$.

### Proof

When $n-B <0$, we study the topological type of endemic equilibrium $(x^{*},y^{*})$ and stability.

From (9) at $(x^{*},y^{*})$, we have the Jacobian matrix

$$ M_{1}= \begin{pmatrix} \frac{B-2(1+p)x^{*}-n}{1+q} & \frac{-x^{*}}{1+q} \\ p & -1 \end{pmatrix}, $$

where

$$\begin{aligned} \det (M_{1}) =&\frac{2(1+p)x^{*} +n-B}{1+q}+\frac{px^{*}}{1+q} \\ =&\frac{(1+p)x^{*}+n-B+px^{*}}{1+q}. \end{aligned}$$

Thus $\det (M_{1})$ has not a unique sign due to

12 $$ S_{1} \stackrel{\Delta }{=}(1+p)x^{*}+n-B+pX^{*}. $$

Relation (12) tells that $S1 > 0 $ yields $\det (M1) > 0$ and $(x^{*},y^{*})$ is a node (focus or center). Also, for the stability of $(x^{*},y^{*})$, one can find the given results. □

### Theorem 3

*The equilibrium*$(x^{*},y^{*})$*of system* (9) *is locally stable in a unique way*, *and also it has a stable node if*$n-B<0$.

### Proof

From $\operatorname{tr}(M_{1})$ we examine $(x^{*},y^{*})$ for stability as follows:

$$\begin{aligned} \operatorname{tr}(M_{1} ) =&\frac{B-2(1+p)x^{*}-n}{1+q} - 1 \\ =&\frac{B-2(1+p)x^{*}-n-1-q}{1+q}. \end{aligned}$$

To determine $\operatorname{tr}(M_{1})$ in sign, we take

$$ S_{2}=-\bigl(2(1+p)x^{*}+q+1+n-B\bigr). $$

Let $S_{2}=0$. Then $n-B<0$. Therefore $S_{2}\neq 0$, which gives that $\operatorname{tr}(M1)\neq 0$. Therefore, for any positive values of parameters, $n-B<0$ does not change the stability of $(x^{*},y^{*})$. Let $p=1$, $q=1$, and $B=1$, which implies that $\operatorname{tr}(M1)=-1<0$ due to the continuity of $\operatorname{tr}(M_{1})$ corresponding to parameters as $\operatorname{tr}(M1)<0$ for $n-B<0$. □

The following theorem summarizes the results for the stability of the original system (1) in terms of the basic reproduction number.

### Theorem 4

*From* (2) *we define*$R_{0}$.

(i) *If*$\mathcal{R}_{0}<1$, *then model* (1) *has a unique disease*-*free equilibrium*$E_{0}=(\frac{a}{\mu },0,0)$, *which is a global attractor in the* 1*st octant*.
(ii) *If*$\mathcal{R}_{0}=1$, *then model* (1) *has a unique disease*-*free equilibrium*$E_{0}=(\frac{a}{\mu },0,0)$*which is an attractor of all orbits in the interior of the* 1*st octant*.
(iii) *If*$\mathcal{R}_{0}>1$, *then model* (1) *has two equilibria*, *a disease*-*free equilibrium*$E_{0}=(\frac{a}{\mu },0,0)$*and an endemic equilibrium*$E^{*}(t)=(S^{*}(t),I^{*}(t),R^{*}(t))$. *The endemic equilibrium*$E^{*}(t)$*is a global attractor in the interior of the* 1*st octant*.

## Numerical results and discussion

We present numerical simulation for system (6) with the used values. We take two different sets of values for immigration parameters involved in model (6) and real data of Wuhan city of China to simulate the results.

According to the NSFD scheme, the first equation of our considered model (6) may be expressed as

13 $$ \frac{dS(t)}{dt}=a -CI(t)S(t) \bigl(1+\gamma I(t)\bigr) - \mu S(t) +\alpha R(t), $$

which is decomposed in the NSFD scheme as follows:

14 $$ \frac{S_{j+1}-S_{j}}{h}=a -CI_{j}(t)S_{j}(t) \bigl(1+\gamma I_{j}(t)\bigr) -\mu S_{j}(t)+ \alpha R_{j}(t). $$

Like (14), we can decompose model (6) in the NSFD scheme and write the whole system as follows:

15 $$\begin{aligned}& S_{j+1} = S_{j}+h \bigl(a -CI_{j}(t)S_{j}(t) \bigl(1+\gamma I_{j}(t) \bigr) -\mu S_{j}(t)+ \alpha R_{j}(t) \bigr), \\& I_{j+1} = I_{j}+h \bigl(CI_{j}(t)S_{j}(t) \bigl(1+\gamma I_{j}(t)\bigr)+(\mu + \beta +\delta )I_{j}(t) \bigr), \\& R_{j+1} = R_{j}+h \bigl(\beta I_{j}(t) -( \alpha +\mu )R_{j}(t) \bigr). \end{aligned}$$

**Figure 2:**
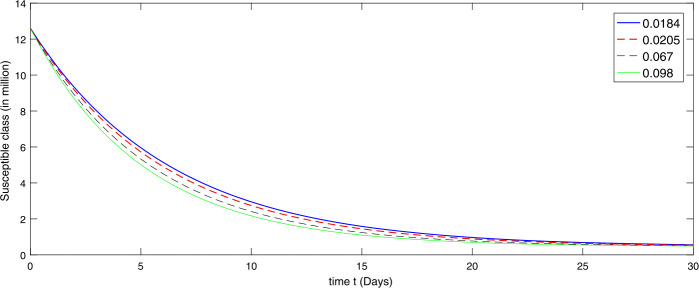
Dynamical behavior of the susceptible class in the presence of given rate of emigrant(s) as Case I from 10 February to 10 March (2020)

**Figure 3:**
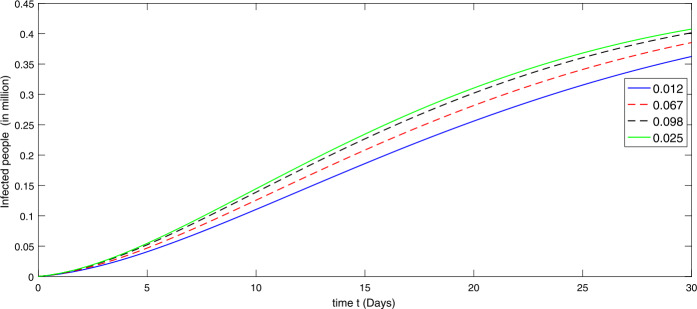
Dynamical behavior of the infected class in the presence of given rate of emigrant(s) as Case I from 10 February to 10 March (2020)

**Figure 4:**
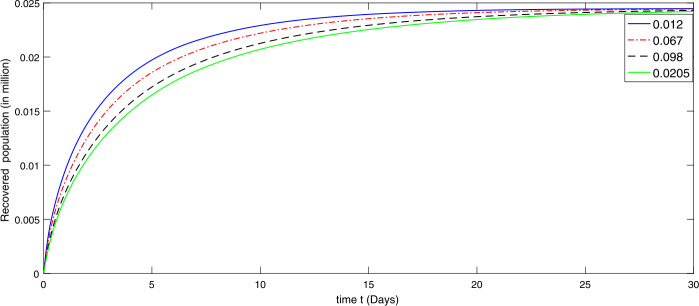
Dynamical behavior of the recovered class in the presence of given rate of emigrant(s) as Case I from 10 February to 10 March (2020)

Using the scheme developed in (15), we present the numerical simulation of the model corresponding to the given values. In the presence of given rate of emigrant(s) in Case I as $[ 0.098, 0.067, 0.0205, 0.0184]$, we present by graph according to the given data in Figs. 2–4 to investigate the transmission dynamics of the various compartments of the considered model.

During the first thirty days in the presence of excessive rate of immigration the susceptible population is decreasing as shown in Fig. 2. When the immigration rate is high, the decline in the population of uninfected (susceptible) people is observed, because they are exposed to infection. Hence the higher the immigration rate, the faster the growth rate of the infected population and vice versa. As a result, more deaths will occur along with the recovery from the disease. Therefore the growth in the recovery class is also different against different immigration. The concerned dynamics are presented by Figs. 3 and 4, respectively.

**Figure 5:**
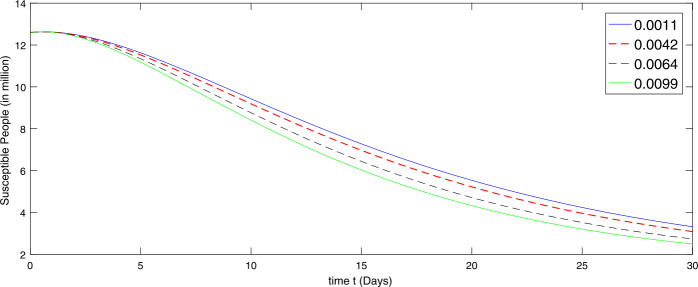
Dynamical behavior of the susceptible class in the presence of given rate of emigrant(s) as Case II from 10 March to 10 April (2020)

**Figure 6:**
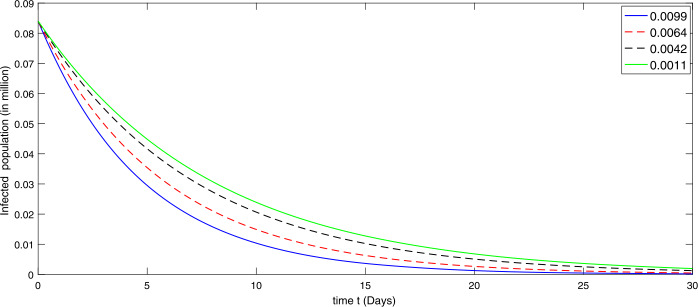
Dynamical behavior of the infected class in the presence of given rate of emigrant(s) as Case II from 10 March to 10 April (2020)

Further we present by graphs in Figs. 5–6 the dynamical behavior of the transmission dynamics corresponding to the second set of values of immigration rate assumed as $[ 0.0099, 0.0064, 0.0042, 0.0011]$ as Case II.

**Figure 7:**
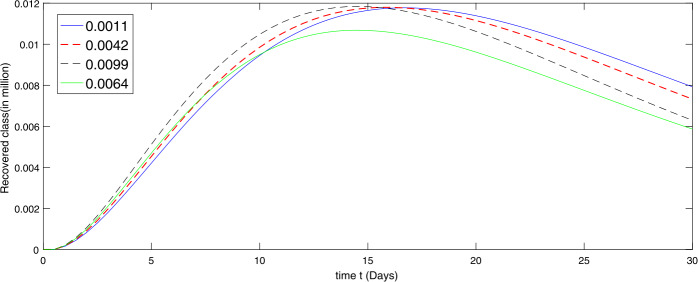
Dynamical behavior of the recovered class in the presence of given rate of emigrant(s) as Case II from 10 March to 10 April (2020)

We see that the immigration slightly reduced during the thirty days from 10 March to 10 April. The decline in the susceptible population at different rate is shown by Fig. 5, while the corresponding dynamics of the infectious and recovered classes are presented via Figs. 6 and 7, respectively. As the immigration rate is decreasing, the susceptibility is decreasing at rapid speed, and consequently the infection rate is going down. The recovered population is also growing with faster speed when immigration rate is low, because the chance of catching infection is decreasing.

## Conclusion

A mathematical model addressing the current novel COVID-19 under three compartments, susceptible, infected, and recovered, has been studied. By nonlinear analysis the existence of global and local stability analysis has been demonstrated. On using nonstandard finite difference numerical method, we have simulated the results by using the real data of Wuhan city during the last sixty days from 10 February 2020 to 10 April 2020. Our model has been simulated for the fixed values of the parameters except immigration rate. In the first set of data we have simulated the model against the highest values of immigration rate. We observed that due to this the infection has been rapidly transmitted from person to person during the first thirty days in the mentioned place. During this time more deaths occurred and the recorded rate also increased accordingly. After reducing the concerned immigration rate properly, the dynamics was greatly affected and the infection rate started decreasing, and the recovery rate also increased with different rate because the rate of immigration was different. With smaller immigration rate, the rate of spread of infection is slow as compared at higher order and vice versa. We concluded that minimizing the immigration during this outbreak can cause the increase in protection rate. In other words avoiding unnecessary immigration of people will greatly help in reducing or controlling this disease. Therefore this model is an indication for further study in this area.

## Abbreviations

NSFD: Nonstandard finite difference scheme
COVID-19: Coronavirus-19 infectious disease

## References

[1] World Health Organization (WHO): Naming the coronavirus disease (COVID-19) and the virus that causes it. Archived from the original on 28 February 2020. Retrieved, 28 February; 2020

[2] Coronavirus (COVID-19) Mortality Rate (2020). www.Worldometers.info

[3] Wu J.T., Leung K., Leung G.M. (2020). Nowcasting and forecasting the potential domestic and international spread of the 2019-nCoV outbreak originating in Wuhan, China: a modelling study. Lancet.

[4] Zhao S. (2020). Preliminary estimation of the basic reproduction number of novel coronavirus (2019-nCoV) in China, from 2019 to 2020: a data-driven analysis in the early phase of the outbreak. Int. J. Infect. Dis..

[5] Abdo M.S. (2020). On a comprehensive model of the novel coronavirus (COVID-19) under Mittag-Leffler derivative. Chaos Solitons Fractals.

[6] Ming, W.K., Huang, J., Zhang, C.J.: Breaking down of healthcare system: mathematical modelling for controlling the novel coronavirus (2019-nCoV) outbreak in Wuhan. BioRxiv, China (2020)

[7] Chan J.F.-W., Kok K.-H., Zhu Z. (2020). Genomic characterization of the 2019 novel human-pathogenic coronavirus isolated from patients with acute respiratory disease in Wuhan, Hubei, China. Emerg. Microbes Infect..

[8] Worldometers. Coronavirus cases (2020). https://www.worldometers.info/coronavirus/coronavirus-cases/ (accessed 1.05.20)

[9] Yousaf M., Zahir S., Riaz M., Hussain S.M., Shah K. (2020). Statistical analysis of forecasting COVID-19 for upcoming month in Pakistan. Chaos Solitons Fractals.

[10] Zhao S. (2020). Estimating the unreported number of novel coronavirus (2019-nCoV) cases in China in the first half of January 2020: a data-driven modelling analysis of the early outbreak. J. Clin. Med..

[11] Zhou P. (2020). A pneumonia outbreak associated with a new coronavirus of probable bat origin. Nature.

[12] Gao W. (2020). Novel dynamic structures of 2019-nCoV with nonlocal operator via powerful computational technique. Biology.

[13] Goel N.S. (1971). On the Volterra and other nonlinear models of interacting populations. Rev. Mod. Phys..

[14] Chen T.-M. (2020). A mathematical model for simulating the phase-based transmissibility of a novel coronavirus. Infect. Dis. Poverty.

[15] Hui D.S., Azhar E.I., Madani T.A. (2020). The continuing 2019-nCoV epidemic threat of novel coronaviruses to global health—the latest 2019 novel coronavirus outbreak in Wuhan, China. Bull. Math. Biol..

[16] Gumel A.B. (2004). Modelling strategies for controlling SARS out breaks. Proc. R. Soc. Lond. B, Biol. Sci..

[17] Rahman G. (2018). Host vector dynamics of pine wilt disease model with convex incidence rate. Chaos Solitons Fractals.

[18] Atangana A. (2020). Fractional discretization: the African tortoise walk. Chaos Solitons Fractals.

[19] Kumar D., Singh J., Al-Qurashi M., Baleanu D. (2019). A new fractional SIRS-SI malaria disease model with application of vaccines, anti-malarial drugs, and spraying. Adv. Differ. Equ..

[20] Chen Y., Guo D. (2016). Molecular mechanisms of coronavirus RNA capping and methylation. Virol. Sin..

[21] Ge X.Y. (2013). Isolation and characterization of a bat SARS-like coronavirus that uses the ACE2 receptor. Nature.

[22] Shah K. (2020). Semi-analytical study of pine wilt disease model with convex rate under Caputo–Fabrizio fractional order derivative. Chaos Solitons Fractals.

[23] Danane J., Allali K., Hammouch Z. (2020). Mathematical analysis of a fractional differential model of HBV infection with antibody immune response. Chaos Solitons Fractals.

[24] Khan M.A., Hammouch Z., Baleanu D. (2019). Modelling the dynamics of hepatitis E via the Caputo–Fabrizio derivative. Math. Model. Nat. Phenom..

[25] Atangana A. (2020). Modelling the spread of COVID-19 with new fractal-fractional operators: can the lockdown save mankind before vaccination?. Chaos Solitons Fractals.

[26] Lin Q. (2020). A conceptual model for the coronavirusdisease 2019 (COVID-19) outbreak in Wuhan, China with individual reaction and governmental action. Int. J. Infect. Dis..

[27] Khan M.A., Atangana A. (2020). Modeling the dynamics of novel coronavirus (2019-nCov) with fractional derivative. Alex. Eng. J..

[28] Ndäirou F. (2020). Mathematical modeling of COVID-19 transmission dynamics with a case study of Wuhan. Chaos Solitons Fractals.

[29] Worldometers. Coronavirus cases (2020). https://www.worldometers.info/coronavirus/coronavirus-cases/ (accessed 2.04.20)

[30] Manning, P.M., Margrave, G.F.: Introduction to non-standard finite-difference modelling. CREWES Research Report 18, 10 pages (2006)

[31] Lu R., Zhao X., Li J., Niu P., Yang B., Wu H., Tan W. (2020). Genomic characterisation and epidemiology of 2019 novel coronavirus, implications for virus origins and receptor binding. Lancet.

[32] Buonomo B., Lacitignola D. (2008). On the dynamics of an SEIR epidemic model with a convex incidence rate. Ric. Mat..

